# Ownership and technical efficiency of hospitals: evidence from Ghana using data envelopment analysis

**DOI:** 10.1186/1478-7547-12-9

**Published:** 2014-04-08

**Authors:** Caroline Jehu-Appiah, Serufusa Sekidde, Martin Adjuik, James Akazili, Selassi D Almeida, Frank Nyonator, Rob Baltussen, Eyob Zere Asbu, Joses Muthuri Kirigia

**Affiliations:** 1 African Development Bank, OSHD.3, BP323, Tunis, Belvedere, Tunisia; 2 Oxford Policy Management, Oxford, UK; 3 Navrongo Health Research Center, Navrongo, Ghana; 4 World Health Organization, Accra, Ghana; 5 Ministry of Health, Accra, Ghana; 6 Radboud University, Nijmegen, Netherlands; 7 Health Authority, Abu Dhabi, United Arab Emirates; 8 World Health Organization Regional Office for Africa, Brazzaville, Congo

**Keywords:** Data envelopment analysis, Technical efficiency, Scale efficiency, Hospitals, Ownership

## Abstract

**Background:**

In order to measure and analyse the technical efficiency of district hospitals in Ghana, the specific objectives of this study were to (a) estimate the relative technical and scale efficiency of government, mission, private and quasi-government district hospitals in Ghana in 2005; (b) estimate the magnitudes of output increases and/or input reductions that would have been required to make relatively inefficient hospitals more efficient; and (c) use Tobit regression analysis to estimate the impact of ownership on hospital efficiency.

**Methods:**

In the first stage, we used data envelopment analysis (DEA) to estimate the efficiency of 128 hospitals comprising of 73 government hospitals, 42 mission hospitals, 7 quasi-government hospitals and 6 private hospitals. In the second stage, the estimated DEA efficiency scores are regressed against hospital ownership variable using a Tobit model. This was a retrospective study.

**Results:**

In our DEA analysis, using the variable returns to scale model, out of 128 district hospitals, 31 (24.0%) were 100% efficient, 25 (19.5%) were very close to being efficient with efficiency scores ranging from 70% to 99.9% and 71 (56.2%) had efficiency scores below 50%. The lowest-performing hospitals had efficiency scores ranging from 21% to 30%.

Quasi-government hospitals had the highest mean efficiency score (83.9%) followed by public hospitals (70.4%), mission hospitals (68.6%) and private hospitals (55.8%). However, public hospitals also got the lowest mean technical efficiency scores (27.4%), implying they have some of the most inefficient hospitals.

Regarding regional performance, Northern region hospitals had the highest mean efficiency score (83.0%) and Volta Region hospitals had the lowest mean score (43.0%).

From our Tobit regression, we found out that while quasi-government ownership is positively associated with hospital technical efficiency, private ownership negatively affects hospital efficiency.

**Conclusions:**

It would be prudent for policy-makers to examine the least efficient hospitals to correct widespread inefficiency. This would include reconsidering the number of hospitals and their distribution, improving efficiency and reducing duplication by closing or scaling down hospitals with efficiency scores below a certain threshold. For private hospitals with inefficiency related to large size, there is a need to break down such hospitals into manageable sizes.

## Background

The pursuit of efficiency has become the central objective of policy makers within most health systems [1]. This is much more evident in Africa where the ability to adequately meet health care needs is exacerbated by extensive inefficiencies, especially within the hospital sector [2-8]. Since the year 2000, fourteen African countries have undertaken health facility efficiency studies to guide them in the development of interventions to reduce waste of scarce resources. These studies demonstrate that DEA is an important tool for policy advice [9-13]. Apart from the one study carried out in Zambia, none of these studies assessed the efficiency of hospitals by ownership type [5]. The type of ownership of a hospital plays a relevant role in explaining economic performance since different ownership structures create different incentives to economic actors [14]. Because of this, the question of whether economic behaviour is affected by ownership type and how it does so has been of longstanding interest to researchers [15].

Ghana’s population of 24.97 million is served by a total of 3,220 health facilities, of which 1,607 are government owned, 91 are quasi-government, 245 are owned by the faith-based organizations and 1, 277 are private-for-profit. Out of the total number of health facilities, there are 3 teaching hospitals, 3 psychiatric hospitals, 9 regional hospitals, 343 district hospitals and 2,094 centres, clinics and poly-clinics. Forty-four percent of the hospitals are private-for-profit while the Ghana Health Services owns 31% of the total hospitals in the country and the Christian Health Association of Ghana owns 16% of the hospitals. Six percent of the hospitals are quasi-government while 3% of the hospitals are Islamic-owned [16].

One of the pillars of the Ghana health sector reforms has been the improvement of efficiency in service delivery. The decentralization of health systems seems not to have translated into improved efficiency and productivity so, in practice, much remains to be done. Marked variations exist in regional performance and there are still pockets of low productivity and wastage. Prior research using DEA identified almost half (47%) of a sample of public district hospitals to be inefficient [17]. Greater promotion of accountability and ensuring value for money is required as there is room for improving efficiency in our hospitals.

Our study draws on Ghanaian hospital data for 2005 to explore the technical efficiency of public, mission and private hospital efficiency at that time, and to demonstrate how a study of hospital efficiency can inform decision-making. We address three research questions: Were the government, mission, private and quasi-government district hospitals in Ghana relatively technically efficient? What were the magnitudes of output increases and/or input reductions needed for inefficient hospitals to operate relatively efficiently? How was the efficiency score for each hospital correlated to ownership?

The specific objectives of our study were: (a) to estimate the relative technical and scale efficiency of government, mission, private and quasi-government district hospitals in Ghana in 2005; (b) to estimate the magnitudes of output increases and/or input reductions that would have been required to make relatively inefficient hospitals more efficient; and (c) to use Tobit regression analysis to estimate the impact of ownership on hospital efficiency.

## Data and methods

### Sampling and data collection

From an initial sample of 167 district hospitals, complete data was available for 128 district hospitals in the variables required for the analysis. The final list of 128 hospitals comprised of 73 public hospitals, 42 mission hospitals, 7 quasi-government hospitals and 6 private hospitals distributed across all the 10 regions of the country.

We used the Ghana Health Service (2000) definition for district hospitals, which are hospitals that provide a full range of outpatient and inpatient services and may not necessarily be the only hospital in the district but have to be located in the district capital [18]. Essential services provided include: medicine, surgery, obstetrics and gynaecology and paediatrics. Essential support for clinical services include: anaesthesia, diagnostic imaging (radiology/ultrasound), clinical laboratory and rehabilitation.

The entire population of designated public district hospitals and mission district hospitals was included in the study. However the private and quasi-government hospitals were purposively sampled using a service availability mapping tool to ensure they delivered similar services to the public district hospitals.

The data set for this study was collected for the financial year period 2005 using a questionnaire adapted from the WHO Regional Office for Africa. Twelve trained enumerators collected data from each hospital. Filled questionnaires had to be signed by the hospital-in-charge to ensure validity. Data was collected on 11 types of inputs and 10 outputs. Based on completeness of data the final selection was limited to 4 inputs and 4 outputs. As shown in Table 1, inputs included total recurrent expenditures, number of clinical staff, number of nonclinical staff, and number of beds. Human resources were classified into clinical and nonclinical staff. The total recurrent expenditure was inclusive of salaries of personnel, expenditure on drugs and expenditure on other goods and services. Hospital outputs were categorized as the annual total of outpatient visits, inpatient days, deliveries, laboratory test.

**Table 1:** Definition and measurement of variables

**Variables**	**Measurement**
**Inputs**	
Beds	Total number of beds
Clinical staff	Total number of doctors, nurses, pharmacists, medical assistants, physiotherapists etc.
Nonclinical staff	Total number of administrators, orderlies, accountants , nutrition officers etc.
Expenditure	Total recurrent expenditure inclusive of salaries of personnel, expenditure on drugs and expenditure on other goods and services.
**Outputs**	
Inpatient days	Total annual number of inpatient days
Outpatient visits	Annual total number of outpatient visits
Deliveries	Annual total number of deliveries
Laboratory Services	Annual total number of laboratory tests

### Data analysis and assumptions

The data was analysed in a two-stage process. In the first stage the technical efficiency scores were estimated for all the district hospitals using Data Envelopment Analysis (DEA). In the second stage, different levels of ownership as explanatory variables were regressed on efficiency scores to find out if ownership had an effect on the technical efficiency of hospitals.

### Data envelopment analysis (DEA)

DEA is based on relative efficiency concepts proposed by Farrell [19]. Charnes extended and developed Farrell’s approach assuming constant returns to scale (CRS) as a sensitive model for measuring technical efficiency [20]. Following their work, a second DEA model, which assumes variable returns to scale (VRS), was developed to separate pure technical efficiency from scale efficiency [21]. Technical efficiency (TE) refers to the ability of a decision making unit to produce maximum output that is feasible from a given level of inputs (i.e. maximizing output from a given level of inputs). When using input orientation, TE may be defined as minimizing input/resource use for a given level of outputs. The size of a hospital may sometimes be a cause for inefficiency. This is referred to as scale inefficiency and takes two forms – decreasing returns to scale and increasing returns to scale. A hospital may be too large for the volume of activities that it is conducting; and therefore may experience *diseconomies of scale*. On the other hand, a hospital may be too small for its level of operation, and thus experience *economies of scale*.

DEA accommodates multiple inputs and multiple outputs in a single measure of efficiency and has become the dominant approach to efficiency measurement in health care and in many other sectors of the economy [22]. While the parametric approach is guided by economic theory, DEA is a data-driven approach. The location and shape of the efficiency frontier is determined by the data. The construct of the frontier is based on ‘best observable practice’ and is therefore only an approximation to the true unobserved efficiency frontier. In other words, it can tell you how efficient you are compared to your peers but not compared to a ‘theoretical’ maximum. This problem can, however, be minimized by using a large sample and data set.

Data envelopment analysis uses linear programming techniques to compute the efficiency scores of each hospital. Hospitals that are technically efficient (producing on the frontier) have a score of 1 or 100%, whereas inefficient hospitals have efficiency scores of less than 1 (i.e. less than 100%). DEA has the following main advantages:

(i) It easily accommodates multiple inputs and outputs without the requirement for a common denominator of measurement. This makes it particularly suitable for analysing the efficiency of hospitals that use many inputs to produce many outputs, and where it is sometimes difficult to assign prices to many of their outputs.

(ii) It provides specific input and output targets that would make an inefficient hospital relatively efficient. Furthermore, it identifies efficient “peers” for those hospitals that are not efficient. This helps the inefficient hospitals to emulate the functional organization of their peers so as to improve their efficiency.

(iii) It helps to identify both the levels and sources of inefficiency, thus providing guidance on remedial actions to be taken.

However, like many empirical methods, DEA has the following main limitations:

(i) DEA produces results that are very sensitive to measurement error, especially in small samples. For example, if one hospital’s inputs are understated or its outputs overstated, it can become an outlier and significantly reduce the efficiency of other hospitals.

(ii) DEA measures efficiency relative to the best practice within hospitals in the particular sample. Therefore, it is not possible to compare how district hospitals in Ghana fare relative to their counterparts in other countries with respect to technical efficiency.

(iii) The exclusion of an important output or input can bias results and underestimate efficiency.

### First stage analysis

We assumed an output-oriented model with Variable Returns to Scale (VRS) to estimate the efficiency score for each hospital using DEA. The VRS model was adopted under the assumption that in practice there are important economies and diseconomies of scale and not all hospitals are operating at an optimal scale. The choice of using an output-oriented model was guided by the fact that most public and mission hospitals have a more or less fixed quantity of inputs and managers have more managerial flexibility in controlling outputs. Even when inputs such as beds and staff are underutilized, it is not within their power to dispose of them. All hospitals with at least one missing value in any of the output or input variables were omitted from the analysis so as to ensure that the methodological requirements of DEA were met.

The VRS model measured the pure technical efficiency and scale efficiency for each of the sample hospitals. From the VRS model, we analysed whether a hospital’s production indicated increasing return to scale, constant return to scale, or decreasing return to scale by the sign of the variable w. Increasing returns to scale exists if the value of wk is greater than zero (wk > 0), constant returns to scale if the value of wk is equal to zero (wk = 0), and decreasing returns to scale if the value of wk is less than zero (wk < 0). Thus, we can analogize the existence of economies of scale similar to ray economies of scale, confirm the most productive scale size (minimum efficient scale) of a hospital and estimate the number of hospitals operating at the efficient scale.

MaxEk=∑ut+Yrk+wkr=1,....,s

s.t.∑viXik=1,i=1,…,m

∑urYrj−∑viXij+wk≤0,j=1,....,n

v1,…,vs>0

u1,…,um>0

Assuming that there are *j* district hospitals, each with *n* hospital inputs and *m* hospital outputs, the relative efficiency score of a given hospital (*θ*_*0*_) is obtained by solving the following output-orientated CCR DEA linear programming model:

$$\max\;\theta \left(u,v\right)=\left(\frac{\sum_{m=1}^{M}\mu_{m}y_{\mathrm{mk}}}{\sum_{n=1}^{N}v_{n}x_{\mathrm{nk}}}\right)$$

$$\begin{array}{c} \mathrm{Subject}\;\mathrm{to}:1\geq \left(\frac{\sum_{m=1}^{M}\mu_{m}y_{\mathrm{mj}}}{\sum_{n=1}^{N}v_{n}x_{\mathrm{nj}}}\right);\forall j\\ \sum_{n=1}^{N}v_{n}x_{\mathrm{nk}}=1\\ u_{m,}v_{n},y_{\mathrm{mj}},x_{\mathrm{nj}}>0;\forall m,n,j \end{array}$$

Where:

*θ*_*0*_ = the efficiency score of hospital 0;

*X*_*nj*_ = the amount of health system input *n* utilized by the *j*^*th*^ hospital;

*Y*_*mj*_ = the amount of health system output *m* produced by the *j*^*th*^ hospital;

*u*_*m*_ = weight given to health system output *m*;

*v*_*n*_ = weight given to output *n*

In this study there were j Decision Making Units (DMUs); that is, j district hospitals, to be evaluated (j = 1,…, 128). Each DMU consumed varying amounts of n different inputs (n = 1, …,4) to produce m different outputs (m = 1, … ,4). Thus, for example, if DMUj consumes amount xnj of input n and produces ymj of output m. For all DMUs, um is the weight by which each ymj is multiplied, and vn is the weight by which each xnj is multiplied. The DMU that is the target of a given evaluation is designated DMUk’, and it is compared to all j of the DMUs including itself. The analysis software/program maximizes the ratio of weighted outputs to the weighted inputs. The value of the ratio, θ, is the efficiency score of DMUk’ where 0 ≤ θ ≤ 1. A fully efficient DMU receives a score of 1.

We used jackknife analysis to test for the robustness of the DEA technical efficiency measures. This technique helped to assess if there were extreme outliers, which affected the frontier and efficiency scores. In conducting the jackknife analysis, a limited number of samples are obtained by omitting one observation at a time [23]. In our case, we dropped each efficient hospital one at a time from the analysis and efficiency scores re-estimated. We tested the similarity of the efficiency rankings between the model with all the hospitals included and those based on dropping each efficient hospital one at a time using the Spearman rank correlation coefficient. The efficiency scores obtained were robust as indicated by Spearman rank correlation coefficient, which was very close to one.

### Second stage data analysis: the Tobit model

If efficiently operating hospitals have certain common characteristics, this allows for identification of possible causes of inefficiency. Thus in the second stage of the analysis, having calculated the efficiency score, we regressed ownership as an explanatory variable on the efficiency score to find out its effect on the technical efficiency of hospitals.

Using the VRS efficiency score as a dependent variable and given that the scores are right-censored (i.e. upper limit of 100 per cent), a Tobit regression model was used to estimate the adjusted efficiency scores for each hospital. Since, by definition, the DEA scores take on values between 0 and 1, and since some of the data tend to concentrate on these boundary values (i.e., censored at 1), the regression cannot be estimated by ordinary least squares. Therefore, some empirical studies use the Tobit model [24,25]. In our study, we did a univariate analysis where the VRS efficiency score was regressed on the ownership explanatory variable.

The Tobit obtains estimates of the linear Tobit model, where the dependent variable is either zero or positive. The method used is maximum likelihood under the assumption of homoskedastic normal disturbances. The standard Tobit model involves truncation of the dependent variable below zero. For this study, the efficiency score was censored at 100% (upper limit) and so an upper limit of 100% was specified in the model.

The following Tobit regression Model was used:

$$\mathrm{Tobit}\left(\mathrm{yj}\right)=\alpha 0+\alpha 1\mathrm{xj}1+\alpha 2\mathrm{xj}2+\alpha 3\mathrm{xj}3+\ldots +\epsilon j$$

The yj is the constant return to scale efficiency score for the jth hospital, the xj are the explanatory variable, α is the coefficient whose values cannot be interpreted but whose signs are helpful for this study, and the ϵj are the disturbance term assumed to be normally distributed with mean μ and standard deviation σ. We estimated the Tobit regression using Stata 10 for Windows [26].

## Results

### General description

Table 2 below provides a summary of the descriptive statistics from the sample of 128 district hospitals in Ghana. Findings indicate there is some variation in the mean input and output variables by ownership. Whereas the variation in the mean number of beds ranges from 46 in the private district hospitals to 103 beds in the mission district hospitals, the variation for clinical and nonclinical staff inputs differs markedly mostly for private hospitals. The quasi-government hospitals in 2005 saw twice as many outpatient cases as mission and public facilities and thrice as many outpatient cases as the private hospitals.

**Table 2:** Summary of descriptive statistics

**Ownership**	**Num. of Hosp**	**Input variables (means, sd)**			**Output variables (means, sd)**	
		**Beds**	**Clinical**	**Non clinical**	**Outpatient visit**	**Admissions**
Government	73	85 (41.1)	72 (55.4)	47 (40.9)	33,603 (30774.2)	4,056 (2586.4)
Mission	42	104 (48.8)	73 (48.5)	50 (31.4)	31,710 (23459.5)	4,417 (3018.2)
Private	7	46 (8.6)	39 (28.0)	25 (18.3)	18,733 (17522.8)	2,057 (1884.4)
Quasi-Govt	6	69 (23.4)	86 (66.7)	65 (39.5)	78,096 (28034.3)	3,054 (1807.6)

### Efficiency results from the DEA model

#### Technical efficiency scores

**Figure 1:**
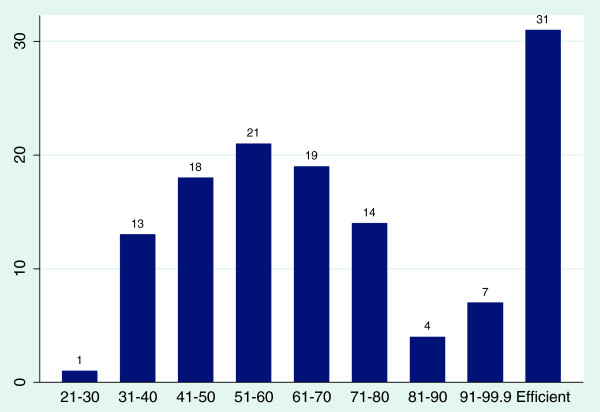
Distribution of scores using the VRS model.

Figure 1 shows the distribution of VRS technical efficiency scores for all the 128 hospitals. Using the VRS model, out of a total of 128 district hospitals, 31 (24.0%) were found to be 100% efficient, 25 (19.5%) were very close to being efficient with efficiency scores ranging from 70 to 99.9% and 71 (56.2%) had efficiency scores below 50%. The lowest performing hospitals had efficiency scores ranging between 21 to 30%.

A summary of average efficiency scores of district hospitals by ownership is provided in Table 3, the mean VRS technical efficiency score for all district hospitals in Ghana is 0.61 or 61%. This indicates a significant amount of inefficiency that is attributable to technical inefficiencies.

**Table 3:** VRS technical efficiency scores

**Ownership**	**N**	**Mean VRS score (%)**	**sd**	**min**	**max**	**Hospitals of Frontier**
Government	73	70.35	22.46	27.4	100	18 (25%)
Mission	42	68.59	23.3	31.92	100	9 (21.42%)
Private	7	55.83	22.72	32.69	100	1 (14%)
Quasi-Govt	6	83	18.15	61.95	100	3 (50%)

Quasi-government hospitals were found to have the highest mean technical efficiency score of 83.9% followed by public hospitals (70.4%), mission hospitals (68.6%) and private hospitals (55.8%). However, some public hospitals also got the lowest individual mean technical efficiency scores of 27.4%, implying they have some of the most inefficient hospitals.

From the total of 128 district hospitals only 31 hospitals (24.2%) are located on the frontier. As shown in Table 3, out of those ‘best practice” hospitals, eighteen are government-owned, nine are mission hospitals, three are quasi-government hospitals and one is a private hospital. Since the quasi-government hospitals have the highest average technical efficiency scores, it is not surprising they have the highest proportion of hospitals on the frontier.

In the second stage analysis, we estimated the effect of ownership on the efficiency of hospitals using a Tobit regression model. The VRS efficiency scores were used as the dependent variable against which explanatory variables were regressed. Tobit analysis results suggest that being a private hospital is a significant factor in determining hospital efficiency (see Table 4).

**Table 4:** Estimation results for Tobit regression model

***Ownership variable***	***Coef.***	***Std. err***	***P value***	***95% Conf. interval***	
Mission	-4.160374	5.227	0.35	(-14.506	6.186)
Private	-23.782	10559	0.002	(-44.681	-2.882)
Quasi-Government	22.514	12.185	0.06	(-1.602	46.630)

Coefficients from Tobit regression analysis are not readily interpretable as effect sizes. Interpretation of these coefficients should focus on the negative or positive sign of the coefficient and whether it is statistically significant or not. While quasi-government ownership is positively associated with hospital technical efficiency, private ownership seems to negatively affect hospital efficiency.

Table 5 below compares the efficiency scores of hospitals by region with mean efficiency scores ranging from a technical efficiency score of 45% in the Volta region to 83% in the Northern Region.

**Table 5:** Mean efficiency score by region

***Region***	***Number of hospitals (N* = *130)***	***Mean VRS score***	***sd***	***min***	***max***
Ashanti	34	61.99	25.6	31.84	100
Brong-Ahafo	13	62.04	17.8	28.3	90.54
Central	10	76.98	20.1	51.05	100
Eastern	14	54.61	20.7	30.56	100
Greater Accra	8	65.04	25.3	29.72	100
Northern	10	83.36	19.8	49.26	100
Upper East	5	72.23	15.7	56.65	92.06
Upper West	5	50.65	29.2	26.49	100
Volta	18	45.02	13.8	22.9	74.39
Western	13	70.01	23.7	30.26	100

District hospitals in the Northern, Central, Western and Upper East regions have average efficiency scores above 70% while the Volta region has the least efficient hospitals with minimum scores of 22%. This may be explained by the fact that the Volta region has one of the largest numbers of health facilities per region/per capita which may be underutilized.

Information on the technical efficiency is more important for management when it is disaggregated by hospitals. Thus, Additional file 1: Tables S1-S4 in the sections below show the situation of each type of hospital included in the study.

#### Technical efficiency of government district hospitals

Seventeen out of a total of 73 government hospitals are 100% efficient (Additional file 1: Table S1). Overall approximately 49% of hospitals had efficiency scores below the average efficiency score for government hospitals. For example, for Kintampo district hospital with a technical efficiency score of 91.6%, Winneba district 66.4% and Lawra hospital 36.7% imply that if these hospitals were to operate efficiently, they are capable of increasing their outputs by 8.4%, 33.6% and 63.5% respectively with the same level of inputs they are currently using. Out of all the government hospitals Peki District hospital in the Volta region obtained the lowest technical efficiency score of 27.4%.

#### Technical efficiency of mission hospitals

From a total of 42 hospitals, 9 (21%) hospitals such as the Catholic hospitals, SDA Wiamoasi, St John of God and St. Francis Xavier, St. Josephs, Baptist Medical center, St. Lukes Kasei and St Peters are on the frontier and are therefore 100% efficient (Additional file 1: Table S2). The West Gonja district hospital in the Northern Region got the lowest efficiency score of 22.9%. This indicates a very high level of inefficiency where the level of outputs could have been increased by 77%. Out of the total sample of mission hospitals 63% operate below the mean efficiency scores for mission hospitals.

#### Technical efficiency of quasi-government hospitals

The quasi-government hospitals seem to exhibit the highest levels of efficiency compared to other hospitals by ownership (Additional file 1: Table S3). Three (50%) out of seven quasi-government hospitals, Kwame Nkrumah University of Science and Technology (KNUST), Trust and Legon University hospitals are on the frontier and are thus 100 efficient. Police hospital, Ghana Ports and Harbour Authority (GPHA) hospital and University hospital in Cape Coast have efficiency scores below the mean of 83.4% for quasi hospitals. The Police hospital had the lowest efficiency scores of 62.0%.

#### Technical efficiency of private hospitals

In Ghana, only a few private hospitals provide clinical services comparable, in scope, to those provided by public district hospitals. Most are equivalent to small clinics and health centres. The initial sample had about 15 private facilities, however only 7 of them were comparable to district hospitals and had complete data required for the analysis. As shown in Table S4, all private hospitals exhibit varying degrees of inefficiency with scores ranging from 32.7% at Atasomanso hospital to 100% at County hospital (Additional file 1: Table S4).

#### Scale efficiency

Variable returns to scale has two dimensions: increasing returns to scale (IRS) and decreasing returns to scale (DRS). When a hospital manifests increasing returns to scale, a one per cent increase in all inputs will be followed by more than one per cent increase in outputs. In contrast, when a hospital exhibits decreasing returns to scale, a percentage increase in inputs will result in less than proportionate increase in outputs. In other words, this denotes the presence of diseconomies of scale. Hospitals that are overall efficient exhibit constant returns to scale and thus have the required optimal size. They are scale efficient.

Table 6 shows 97 (75.0%) of district hospitals in Ghana are scale inefficient. Meaning they are either too small or too large. Increasing returns to scale was the predominant form of scale inefficiency except for private hospitals that show predominantly decreasing returns to scale. Of the 128 hospitals, 67 (52.0%) operate in increasing returns to scale (IRS) implying these hospitals should expand both their inputs and outputs, 31 (24.0%) displayed constant returns to scale (CRS) implying they are operating at their most productive scale sizes and 30 (23.4%) are operating in decreasing returns to scale (DRS). Returns to scale values for each hospital are provided in Table 6.

**Table 6:** **Returns to scale** (**RTS**) **model**

***Ownership***	***Decreasing***	**RTS *constant***	***Increasing***	***N***
Government	17 (22.97%)	17 (22.97%)	39 (53.42%)	73 (100%)
Mission	6 (14.29%)	10 (23.81%)	26 (61.90%)	42 (100%)
Private	6 (85.71%)	1 (14.29%)	0	7 (100%)
Quasi-Government	1 (16.67%)	3 (50%)	2 (33.33%)	6 (100%)
Total	30 (23.44%)	31 (24.22%)	67 (51.34%)	128 (100%)

#### Technical efficiency plotted against selected variables

The graphs shown below are scatter diagrams of all 128 hospitals and selected variables as they relate to efficiency scores. The graphs depict the VRS efficiency scores against the numbers of clinical and nonclinical staff, beds and hospital functional area. This was done to primarily determine the relationship between VRS efficiency scores and the above mentioned variables and to assess their consumption by the most efficient hospitals.

**Figure 2:**
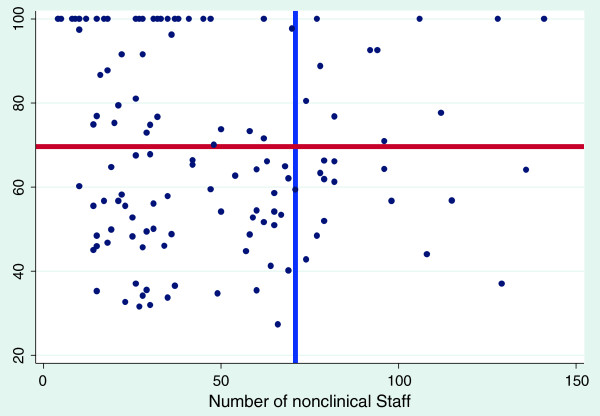
**The effect of non**-**clinical staff on variable return to scale efficiency score blue line represents mean number of nonclinical staff; red line represents mean variable return to scale efficiency score.**

**Figure 3:**
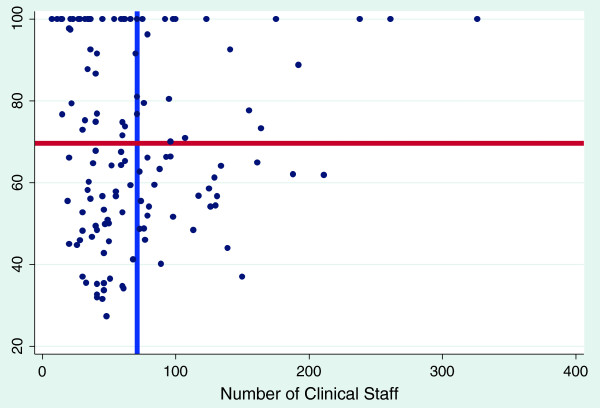
**The effect of clinical staff on variable return to scale efficiency score blue line represents mean number of clinical staff; red line represents mean variable return to scale efficiency score.**

Figures 2 and 3 show that, for clinical and nonclinical staff, the most efficient hospitals (those on the frontier) do not employ more than 100 clinical and 50 non clinical staff to be efficient. One can therefore suggest that district hospitals in Ghana should have a ratio of 2:1 for clinical and non-clinical staff.

**Figure 4:**
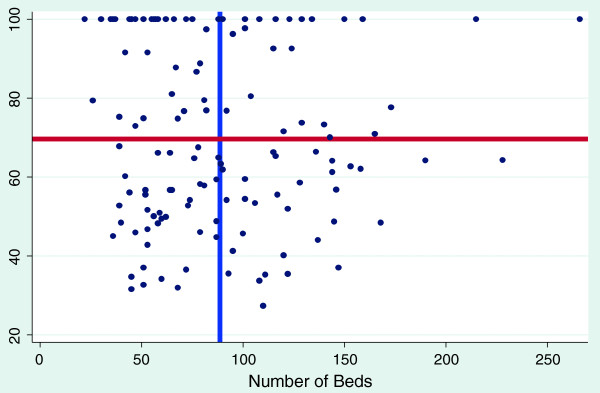
**The effect of number of beds on variable return to scale efficiency score blue line represents mean number of bed; red line represents mean variable return to scale efficiency score.**

Figure 4 plots the efficiency score against the number of beds. The graph demonstrates the huge variations in the number of beds ranging from 40 to 280 beds in our district hospitals. However, the most efficient hospitals have less than 100 beds, on average ranging from 40 to 80 beds. These findings can inform hospital standards on the most appropriate bed size for our district hospitals.

**Figure 5:**
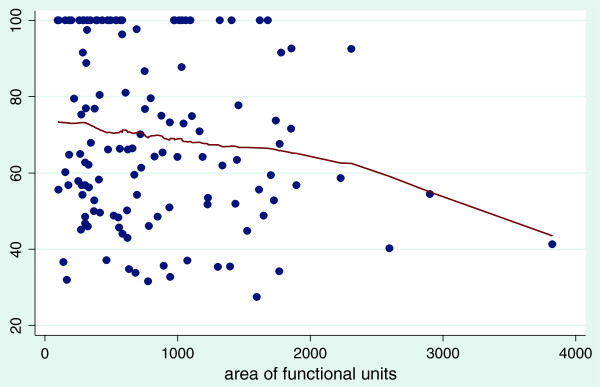
**The effect of the area of functional units in hospitals on variable return to scale efficiency score Bandwidth = 0.8.**

Figure 5 shows that in terms of functional area of our district hospitals, results suggest that efficiency begins to decrease with functional areas above 1100 m^2^ for district hospitals. In other words a hospital with larger space to work does not necessarily confer an increase in its efficiency score.

Figures 2, 3 and 4 also show the mean efficiency scores plotted against mean numbers of clinical, non-clinical staff and bed size. The most efficient hospitals, with scores ranging from above the mean efficiency score to 100%, are found in the top left quadrants. These findings may inform standardization and target setting for district hospitals for the health sector.

#### Potential output improvements

Table 7 provides the output increases expected for public, mission, quasi-government and private hospital with current input levels if hospitals were to operate as efficiently as their peers. In other words, these are the potential gains that should be reaped by the health sector at no extra cost if these inefficient hospitals were to operate efficiently. Results show that to reduce the amount of leakages due to inefficiency the largest output increases are to be made in the private sector, followed by those in mission, public and quasi- government hospitals, in that order

**Table 7:** Potential output improvements per type of hospital

**Output type**	**Actual output**	**Target output if efficient**	**% increase**
Government hospital			
OPD attendance	2,501,596	4,488,118	79%
IPD	304,284	495,785	63%
Deliveries	79,139	130,891	65%
Laboratory services	3,012,825	4,603,862	53%
Mission hospitals			
OPD attendance	1,283,284	2,632,868	105%
IPD	177,408	316,966	79%
Deliveries	34,886	68,789	97%
Laboratory services	1,538,295	2,667,427	73%
Quasi-Government hospitals			
OPD attendance	468580	603,887	29%
IPD	18,329	23,080	29%
Deliveries	4,341	11,708	170%
Laboratory services	249,776	349,084	40%
Private hospitals			
OPD attendance	131,134	298,114	127%
IPD	1,199	21,399	133%
Deliveries	1,075	5,224	386%
Laboratory services	211,345	506,289	140%

## Discussion

To our knowledge this is the first study in Sub-Saharan Africa that covers the whole country, compares district hospitals by ownership and provides empirical evidence on the performance of district hospitals in Ghana. Our findings indicate that overall, approximately 76% of district hospitals were inefficient, and therefore, not using scarce resources optimally. Furthermore, our study suggests that ownership does affect efficiency. It is often argued that the private sector is more efficient than the public sector in the production of health services. This is based on the assumption that the public sector, which are not-for-profit, do not provide the right incentives for managers to optimize the use of resources [27]. However, similar to other studies, we found the opposite to be true, private for profit hospitals exhibited the highest levels of inefficiency compared to public, mission and quasi-government health facilities [28-30]. We elaborate on our findings below.

The quasi-government hospitals were found to be the most efficient with efficiency scores of 83.9% followed by public hospitals (70.4%), mission hospitals (68.59%) and private hospitals (55.8%). Quasi-government facilities are government owned but are autonomously managed which may explain their efficiency.

The majority of ‘best practice” hospitals are government-owned. One explanation may be that since government hospitals operate under significant budget constraints they have to provide medical care at lower costs and, therefore, more efficiently. However, government hospitals also display greater variations in inefficiency scores.

In the literature, the evidence on the impact of ownership on efficiency is mixed. Some studies have found the public sector to be more efficient [28-31]. Others have found the contrary to be true [32]. For some the evidence was inconclusive [33]. In line with our findings, Hollingsworth, in his meta-analysis of 317 publications concludes that public provision of health care services may be potentially more efficient than private [22]. Our findings demonstrate that to be efficient, private facilities would have to increase their outputs two to three-fold, while holding inputs constant. Managers of private hospitals will therefore have to find innovative ways of generating demand for their services, capitalizing on the fact that patients may prefer private hospitals to public hospitals. To deal with the prohibitively high fees, people should be encouraged to enrol with the National Health Insurance Scheme.

Private hospitals in Ghana are accredited and registered by the Private Hospitals and Maternity Homes Board. However, only two of the seven private hospitals in this study are accredited. This is not surprising given that less than 15% of Ghanaian private hospitals are accredited. Yet, the accreditation process provides an opportunity to fulfil basic requirements in terms of staffing norms, equipment and infrastructure in line with set standards which will have a positive impact on efficiency.

Looking at regional performance, district hospitals in the Northern region exhibited the highest mean efficiency score of 83% and the Volta region had the lowest mean score of 43%. A 2008 study of the technical efficiency of health centres in Ghana found the least efficient health centres to be in the Volta region [34]. This may be ascribed to the Volta region being one of the most endowed in terms of number of hospitals per population and the low levels of efficiency may thus be attributable to the excess capacity of hospitals, low outpatient department attendance and low occupancy rates.

Currently, Ghana is in the process of trying to standardize hospitals and needs to determine the most appropriate bed size, equipment, staffing norms and targets for the range of services to be provided for each level. Our study demonstrates that the most efficient district hospitals operate within a range of approximately 50–80 beds. Similarly, for clinical and nonclinical staff the most efficient hospitals employ not more than 100 clinical staff and not more than 50 nonclinical staff. Our findings therefore provide evidence of economies of scale of up to 100 beds for district hospitals and support the conventional view that the larger the hospital the less efficient it will be. In terms of total functional area, not more than 1100 m^2^ appears optimal. Therefore in setting standards for hospitals it is desirable for the ministry of health, in addition to equity considerations, to control the number and size of hospitals in the country based on catchment population, demand and access.

The data is widely dispersed in terms of inputs such as expenditure, hospital beds and staff. This variability again, points to the fact that our district hospitals lack homogeneity. The number of hospitals and beds in Ghana is as a result of series of decisions taken by government, private organization and mission institutions over many years. This has resulted in a pattern where some areas are well served and others are not. It appears from our study that we have more beds than what is required for the given output levels, especially for private hospitals. This does not imply that overall the number of beds exceeds the populations need for services. Hospital bed ratios per 1,000 population in Ghana are low and less than two. This is in contrast to means of more than four beds per 1,000 in middle-income countries and more than eight beds per 1,000 in high-income countries. Given that utilisation of hospital beds is both demand and supply-driven, it is safe to say the current number of beds in district hospitals is in excess of what is required with the current demand levels.

The number of staff (clinical and non-clinical) per bed ranges from 0.7 for government, 0.85 for mission, 0.71 for private and 0.4 for quasi government hospitals. These values are above the international benchmark of 60 staff for 140 bed hospital or under 0.5 staff per bed [35]. Again, this does not mean that Ghana has excess human resource capacity, but that we have staff in the sampled hospitals for the given number of beds and outputs.

With regards to optimal hospital size, most district hospitals (75.0%) are not operating at an optimal size and are thus scale inefficient (bigger or smaller than optimal). Most public hospitals (53.0%) and mission (62.0%) are exhibiting increasing returns to scale. The average cost of production can decrease if the scale of operation increases, meaning efficiency will increase if such hospitals will increase their outputs. This is easier said than done since increasing scale of operations requires an increase in demand for services which to an extent is beyond the control of managers. Policy makers should consider merging of hospitals that are in close to one another.

Eighty five percent (85.0%) of private hospitals, 23% of government hospitals, 14% of mission hospitals and 17% of quasi-government hospitals exhibit decreasing returns to scale. This implies that they are too large and will become scale efficient if they decrease their scale of operations or are downsized. With the new paradigm for health and emphasis on primary care, the option to reallocate resources from secondary care to primary and preventative care should be considered. Given the widespread scale inefficiencies some hospitals could be converted into health centres by downsizing both the services provided and staff composition and numbers [36]. However, it is worth noting that there may be resistance to this from the actors involved and may therefore not be politically feasible.

Finally, we observe that 76% of our hospitals can increase their outputs with the current levels of inputs to operate as efficiently as their peers. However increasing the level of outputs requires an increase in the demand of health care, which may be beyond the control of the hospital manager. Nonetheless, the introduction of national health insurance in Ghana is reducing financial barriers and generating demand for hospital services and may lead to efficiency improvements as demonstrated by a similar study of Korean public and private hospitals [37]. The study identified insurance coverage as a significant factor in improving hospital efficiency. It is therefore important that efforts are made to increase insurance coverage. Specifically for public hospitals, this means explicitly dealing with the negative attitude of their staff towards insured clients.

### Limitations of the study

First, the analysis reported in this paper is based on hospital inputs and outputs data for 2005. Much has happened since 2005, notably in terms of the country’s socioeconomic and health development. Therefore, the results of this analysis are not meant to uncritically feed into current decision-making, but rather to illustrate the potential usefulness of such efficiency analyses.

Second, due to the lack of data, this study did not include the expenditures on pharmaceuticals and non-pharmaceutical supplies among the inputs. Nor does the study take into consideration the differences that may exist between the categories of nurses and doctors in the various hospitals. In addition, even within the same health workforce category, the quality of labour input may vary depending on individual health worker skills, professional experience and health status.

Third, the hospitals were not adjusted for case-mix thereby affecting the interpretation of ranges prescribed for input variables reductions and downsizing of units. Fourth, the Tobit model could not determine which variables most influenced efficiency scores to increase the relevance of the study for management purposes.

Fifth, there has been on-going debate between two schools of thought over the statistical properties of the two-stage DEA estimator. In one school of thought, academics such as Simar and Wilson [38] argue that since DEA output scores are biased and environmental variables are correlated to output and input variables, the conventional statistical inferences are invalid in the second-stage regression, and recommend use of bootstrap methods. In the second school of thought, scholars such as Ramalho et al. [39], McDonald [40] and Ruggiero [41] contend that econometric models such as probit, logit, and truncated regression (Tobit) can be used for second-stage estimation of the impact of environmental variables on efficiency scores. Afonso and Aubyn [42] maintain that “Even if Tobit results are possibly biased, it is not clear that bootstrap estimates are necessarily more reliable, based on a set of assumption concerning the data generation process and the perturbation term distribution that may be distributed (p. 1429)”. In their study, the censored normal Tobit and bootstrap algorithms yielded very similar results. Therefore, since there is no consensus in the literature, we chose to estimate the Tobit model because DEA efficiency scores are bounded between 0 and 1 (or 0% and 100%).

## Conclusions

Given the findings of this study, the Ministry of Health in Ghana and its agencies should carry out reviews of the numbers of district hospitals in Ghana and their distribution. This should be done with a view of improving allocative efficiency between hospitals and regions and reducing duplication by closing down or scaling down hospitals with efficiency scores below a certain threshold. However, in practice, this may be controversial and may face political resistance.

For private hospitals with technical inefficiencies related to large size (decreasing returns to scale) there is a need to break down such hospitals into a manageable size. There is the need to build the capacity of the private sector to manage resources and promote accreditation to ensure basic standards are met.

National insurance schemes, as purchasers of health services, face information asymmetries that do not favour them when negotiating contracts with health providers. They often find it difficult to judge whether providers are offering good value for money. Generating mean efficiency for each hospital will help these national insurance schemes better understand the performance of health providers relative to best practice. In effect, this will introduce elements of “yardstick competition” into the purchasing arrangements.

The health sector should generate demand for its services by improving staff attitude and the quality of care. Strategies to increase national health insurance coverage should be employed to increase demand, improve access and reduce the inefficiencies in hospitals.

Finally, more research on ownership and understanding of organizational decision-making and market-level dynamics can contribute to better understanding of the institutional context in which ownership matters for provider performance. It will help identify which institutional reforms could improve performance, based on best practice.

## Competing interests

The authors declare that they have no competing interests.

## Authors’ contributions

CJA, EZ, SD were involved in the study design and analysis. CJA was responsible for interpretation of results and drafting of the manuscript with critical contributions from SS. JA, RB, FN, JK JMK and EZA provided inputs. All authors read and approved the final manuscript.

## Supplementary Material

Additional file 1: Table S1 Summary of technical efficiency scores of government district hospitals. **Table S2.** Summary of technical efficiency scores of mission district hospitals. **Table S3.** Summary of technical efficiency scores of quasi-government hospitals. **Table S4.** Summary of technical efficiency scores of private hospital. Click here for file

## References

[B1] WHO AFRO Health financing: A strategy for the African Region 2006 Brazzaville

[B2] Kirigia JM Emrouznejad A Cassoma B Asbu EZ Barry S A performance assessment method for hospitals: the case of Municipal Hospitals in Angola J Med Syst 2008 32 509 519 10.1007/s10916-008-9157-5 19058655

[B3] Zere E Mbeeli T Shangula K Mandlhate C Mutirua K Tjivambi B Kapenambili W Technical efficiency of district hospitals: evidence from Namibia using data envelopment analysis Cost Eff Resour Alloc 2006 4 5 10.1186/1478-7547-4-5 16566818 PMC1524815

[B4] Tlotlego N Nonvignon J Sambo LG Asbu EZ Kirigia JM Assessment of productivity of hospitals in Botswana: a DEA application Int Arch Med 2010 3 1 14 10.1186/1755-7682-3-1 21054835 PMC2992505

[B5] Masiye F Investigating health system performance: an application of data envelopment analysis to Zambian hospitals BMC Health Serv Res 2007 7 58 10.1186/1472-6963-7-58 17459153 PMC1878476

[B6] Kirigia JM Emrouznejad A Sambo LG Measurement of technical efficiency of public hospitals in Kenya: using Data Envelopment Analysis J Med Syst 2002 26 39 45 10.1023/A:1013090804067 11777310

[B7] Zere E Mcintyre D Addison T Hospital efficiency and productivity in three provinces of South Africa South Afr J Econ 2005 69 336 358 10.1111/j.1813-6982.2001.tb00016.x

[B8] Kirigia JM Sambo LG Scheel H Technical efficiency of public clinics in Kwazulu-Natal Province of South Africa East Afr Med J 2001 78 3 Suppl S1 S13 12002061 10.4314/eamj.v78i3.9070

[B9] Marschall P Flessa S Assessing the efficiency of rural health centres in Burkina Faso: an application of Data Envelopment Analysis J Public Health 2008 17 87 95

[B10] Ichoku HE Fonta WM Onwujekwe OE Kirigia JM Evaluating the Technical Efficiency of Hospitals in Southeastern Nigeria Eur J Bus Manag 2011 3 24 37

[B11] Kirigia JM Emrouznejad A Vaz RG Bastiene H Padayachy J A comparative assessment of performance and productivity of health centres in Seychelles Int J Prod Perform Manag 2008 57 72 92 10.1108/17410400810841245

[B12] Kirigia JM Sambo LG Renner A Alemu W Seasa S Bah Y Technical efficiency of primary health units in Kailahun and Kenema districts of Sierra Leone Int Arch Med 2011 4 1 14 10.1186/1755-7682-4-1 21569339 PMC3108938

[B13] Kirigia JM Lambo E Sambo L Are public hospitals in Kwazulu-Natal Province of South Africa technically efficient? Afr J Heal Sci 2000 7 25 32 17650022

[B14] Barbetta GP Turati G Zago AM Behavioral differences between public and private not-for-profit hospitals in the Italian National Health Service Health Econ 2007 16 75 96 10.1002/hec.1143 16929498

[B15] Spector WD Selden TM Cohen JW The impact of ownership type on nursing home outcomes Health Econ 1998 7 639 653 10.1002/(SICI)1099-1050(1998110)7:7<639::AID-HEC373>3.0.CO;2-0 9845257

[B16] Ghana Health Services The health sector in Ghana: Facts and Figures 2010 Accra,Ghana: GHS

[B17] Osei D D’ Almeida S George MO Kirigia JM Mensah AO Kainyu LH Technical efficiency of public district hospitals and health centres in Ghana: a pilot study Cost Eff Resour Alloc 2005 3 9 10.1186/1478-7547-3-9 16188021 PMC1253524

[B18] Ghana Health Service Ghana Health Service 2000 Accra,Ghana: GHS

[B19] Farrell MJ The measurement of productive efficiency J R Stat Soc 1957 120 253 290

[B20] Charnes A Clark CT Cooper W Golany B A developmental study of data envelopment analysis in measuring the efficiency of maintenance units in the US air forces Ann Oper Res 2 95 112

[B21] Jacobs R Alternative Methods to Examine Hospital Efficiency: Data Envelopment Analysis and Stochastic Frontier Analysis Health Care Manag Sci 2001 4 2 103 15 10.1023/A:1011453526849 11393739

[B22] Hollingsworth B Wildman J The efficiency of health production: re-estimating the WHO panel data using parametric and non-parametric approaches to provide additional information Health Econ 2003 12 493 504 10.1002/hec.751 12759918

[B23] Efron B The Jackknife, the Bootstrap, and Other Resampling Plans 1982 Philadelphia, Pa: Society for Industrial and Applied Mathematics [CBMS-NSF Regional Conference Series in Applied Mathematics, vol. 38]

[B24] Chilingerian J Exploring why some physicians’ hospital practices are more efficient: taking DEA inside the hospital. data envelopment analysis Theory, Methodology, and Applications 1994 167 193

[B25] Kooreman P Data envelopment analysis and parametric frontier estimation: complementary tools J Health Econ 1994 13 345 346 10.1016/0167-6296(94)90035-3

[B26] Statacorp Stata Statistical Software. Release 10

[B27] Hsu J The relative efficiency of public and private service delivery World Health Report Background Paper 39

[B28] Grosskopf S Valdmanis V 201028 Measuring hospital performance. A non-parametric approach J Health Econ 1987 6 89 107 10.1016/0167-6296(87)90001-4 10312167

[B29] Ozcan YA Luke RD Haksever C Ownership and organizational performance. A comparison of technical efficiency across hospital types Med Care 1992 30 781 794 10.1097/00005650-199209000-00003 1518311

[B30] Rosko MD Impact of internal and external environmental pressures on hospital inefficiency Health Care Manag Sci 1999 2 63 74 10.1023/A:1019031610741 10916603

[B31] Helmig B Lapsley I On the efficiency of public, welfare and private hospitals in Germany over time: a sectoral data envelopment analysis study Heal Serv Manag Res Off J Assoc Univ Programs Heal Adm Hsmc Aupha 2001 14 263 274 10.1177/095148480101400406 11725593

[B32] Steinmann L Zweifel P On the (in) efficiency of Swiss hospitals Appl Econ 2003 35 361 370 10.1080/00036840210167183

[B33] Staat M Efficiency of hospitals in Germany: a DEA-bootstrap approach Appl Econ 2006 38 2255 2263 10.1080/00036840500427502

[B34] Akazili J Adjuik M Jehu-Appiah C Zere E Using data envelopment analysis to measure the extent of technical efficiency of public health centres in Ghana Bmc Int Heal Hum Rights 2008 8 11 10.1186/1472-698X-8-11 PMC2605432 19021906

[B35] World Bank World Development Report 1993 Investing in Health, Volume1 1993 Washington, D.C: The World Bank

[B36] Setting a new health policy for Ghana 2005 Accra, Ghana: Ministry of Health

[B37] Jeon B Kwon S Effect of private health insurance on health care utilization in a universal public insurance system: a case of South Korea Health policy 2013 113 1-2 69 76 10.1016/j.healthpol.2013.05.007 23786992

[B38] Simar L Wilson PW Estimation and inference in two-stage, semi-parametric models of production processes J Econ 2007 136 31 64 10.1016/j.jeconom.2005.07.009

[B39] Ramalho EA Ramalho JJS Henriques PD Fractional regression models for second state DEA efficiency analyses J Product Anal 2010 34 239 255 10.1007/s11123-010-0184-0

[B40] McDonald J Using least squares and tobit in second stage DEA efficiency analyses Eur J Oper Res 2009 197 792 798 10.1016/j.ejor.2008.07.039

[B41] Ruggiero J Cooper WW, Seiford LM, Zhu J Performance evaluation in education: modelling educational production Handbook on Data Envelopment Analysis 2004 Boston: Kluwer Academic Publishers 265 298

[B42] Afonso A Aubyn M Assessing health efficiency across countries with a two-step and bootstrap analysis Appl Econ Lett 2011 18 15 1427 1430 10.1080/13504851.2010.541149

