# Supplementary material for: Ownership and technical efficiency of hospitals: evidence from Ghana using data envelopment analysis
Source: Cost Eff Resour Alloc. 2014 Apr 8;12:9. doi: 10.1186/1478-7547-12-9 (PMC4108084; doi:10.1186/1478-7547-12-9)
Supplement: Additional file 1: Table S1 — Summary of technical efficiency scores of government district hospitals. Table S2. Summary of technical efficiency scores of mission district hospitals. Table S3. Summary of technical efficiency scores of quasi-government hospitals. Table S4. Summary of technical efficiency scores of private hospital. [file 1478-7547-12-9-S1.doc]

Supplementary tables

Table 1: Summary of technical efficiency scores of government district hospitals

* RTS – returns to scale; -1 represents decreasing returns to scale; 0 is constant returns to scale; and 1 indicates increasing returns to scale

Table 2: Summary of technical efficiency scores of mission district hospitals

Table 3: Summary of technical efficiency scores of quasi-government hospitals

Table 4: Summary of technical efficiency scores of private hospital
